# Nocturnal arthropod occurrences across Natura 2000 protected areas of Madeira Island (Portugal)

**DOI:** 10.3897/BDJ.14.e209141

**Published:** 2026-10-07

**Authors:** Elisa S.N. Teixeira, Tânia D. Costa, Cátia Gouveia, Virgílio Vieira, Mário Boieiro, Paulo A.V. Borges

**Affiliations:** 1 Faculdade de Ciências Agrárias e Ambiente, Universidade dos Açores, Mestrado em Gestão e Conservação da Natureza, Terceira, Portugal Faculdade de Ciências Agrárias e Ambiente, Universidade dos Açores, Mestrado em Gestão e Conservação da Natureza Terceira Portugal; 2 SPEA BirdLife – Sociedade Portuguesa para o Estudo das Aves, Funchal, Portugal SPEA BirdLife – Sociedade Portuguesa para o Estudo das Aves Funchal Portugal; 3 Basque Centre for Climate Change (BC3), Leioa, Spain Basque Centre for Climate Change (BC3) Leioa Spain; 4 University of Azores, CE3C—Centre for Ecology, Evolution and Environmental Changes, Azorean Biodiversity Group, CHANGE —Global Change and Sustainability Institute, Faculty of Science and Technology, Rua da Mãe de Deus, 9500-321 Ponta Delgada, Ponta Delgada, Azores, Portugal University of Azores, CE3C—Centre for Ecology, Evolution and Environmental Changes, Azorean Biodiversity Group, CHANGE —Global Change and Sustainability Institute, Faculty of Science and Technology, Rua da Mãe de Deus, 9500-321 Ponta Delgada Ponta Delgada, Azores Portugal; 5 Centre for Ecology, Evolution and Environmental Changes (CE3C) & CHANGE – Global Change and Sustainability Institute, Faculty of Sciences, University of Lisbon, 1749-016 Campo Grande, Lisbon, Portugal Centre for Ecology, Evolution and Environmental Changes (CE3C) & CHANGE – Global Change and Sustainability Institute, Faculty of Sciences, University of Lisbon, 1749-016 Campo Grande Lisbon Portugal; 6 IUCN SSC Atlantic Islands Invertebrate Specialist Group, Angra do Heroísmo, Azores, Portugal IUCN SSC Atlantic Islands Invertebrate Specialist Group Angra do Heroísmo, Azores Portugal; 7 University of Azores, CE3C—Centre for Ecology, Evolution and Environmental Changes, Azorean Biodiversity Group, CHANGE —Global Change and Sustainability Institute, School of Agricultural and Environmental Sciences, Rua Capitão João d’Ávila, Pico da Urze, 9700-042, Angra do Heroísmo, Azores, Portugal University of Azores, CE3C—Centre for Ecology, Evolution and Environmental Changes, Azorean Biodiversity Group, CHANGE —Global Change and Sustainability Institute, School of Agricultural and Environmental Sciences, Rua Capitão João d’Ávila, Pico da Urze, 9700-042 Angra do Heroísmo, Azores Portugal; 8 IUCN SSC Monitoring Specialist Group, Angra do Heroísmo, Azores, Portugal IUCN SSC Monitoring Specialist Group Angra do Heroísmo, Azores Portugal

**Keywords:** Island biodiversity, Lepidoptera, Light pollution, Moths, Species inventory, UV light trap, wine-rope

## Abstract

**Background:**

Artificial light at night (ALAN) is increasingly recognised as an important anthropogenic pressure affecting nocturnal biodiversity, particularly in oceanic islands with high endemism levels. Arthropods, especially moths (Lepidoptera), are key components of nocturnal ecosystems, yet their distribution and abundance patterns in Madeira Island remain insufficiently documented and their responses to ALAN are poorly understood. This study was implemented under the LIFE Natura@night project – Mitigating light pollution in Macaronesia (LIFE20 NAT/PT/001098; https://www.naturaatnight.spea.pt), which aims to assess and mitigate the impacts of artificial light at night on biodiversity and ecosystems across the Macaronesian archipelagos, while supporting evidence-based conservation and management actions.

**New information:**

This dataset presents terrestrial arthropod occurrences collected across Natura 2000 protected areas of Madeira Island during two field campaigns conducted in late summer 2022 and spring 2023. Heath UV light traps were complemented with wine-rope bait traps to maximise the detection of nocturnal arthropods. The dataset comprises 1,939 occurrence records (including 35 absence records), documenting 11,885 individuals belonging to 200 taxa (species and subspecies) from 16 terrestrial arthropod groups and is dominated by Lepidoptera (76.2% of all records). Of the 179 lepidopteran taxa recorded, 63 (35.2%) are endemic to Madeira, 68 (38.0%) are native non-endemic taxa, 38 (21.2%) are introduced and 10 (5.6%) are migratory, highlighting the exceptional conservation value of the surveyed Natura 2000 sites.

Several endemic lepidopteran taxa of particular conservation interest were recorded, including *Blastobasis bassii* Karsholt & Sinev, 2004; *Blastobasis laurisilvae* Karsholt & Sinev, 2004; *Blastobasis wollastoni* Karsholt & Sinev, 2004; *Blepharita inexspectata* Weidlich, 2001; *Herbulotina maderae* Pinker, 1971; *Mythimna serradaguae* Wolff, 1977; *Noctua teixeirai* Pinker, 1971; *Xenochlorodes magna* Wolff, 1977; and *Zelleria wolffi* Klimesch, 1983, many of which are characterised by restricted distributions and were sampled in small numbers. Additional rare endemic species included *Agrotis atrux* (Pinker, 1971); *Carposina anopta* Diakonoff, 1988; *Eupithecia latipennata* Prout, 1914; and *Eupithecia rosai* Pinker, 1962.

The survey provided new insights into the arthropod fauna of Madeira, including two potential new records for the Archipelago [*Blastesthia tessulatana* (Staudinger, 1871) and *Heterotoma planicornis* (Pallas, 1772)], a possible first confirmed record (*Eudonia mercurella* Linnaeus, 1758) and the re-confirmation of a previously doubtful taxon (*Blastobasis phycidella* Zeller, 1839). These findings emphasise the value of continued biodiversity monitoring in improving knowledge of Madeira's terrestrial arthropod diversity. The dataset also documents the occurrence of *Hipparchia maderensis* Baker, 1891, the only diurnal butterfly recorded during the survey.

## Introduction

Arthropods are fundamental components of terrestrial ecosystems, contributing to multiple ecological processes, including herbivory, predation, decomposition, nutrient cycling, pollination and overall ecosystem functioning (Noriega et al. 2018). They regulate food-web dynamics both as consumers and as prey, influencing plant communities, ecosystem productivity and the persistence of higher trophic levels. Despite their ecological importance, nocturnal arthropods remain under-represented in biodiversity monitoring programmes because invertebrate monitoring still suffers from major taxonomic, distributional and abundance data gaps, while nocturnal ecological processes and species interactions remain comparatively understudied (Macgregor et al. 2015, Macgregor and Scott-Brown 2020). This gap is particularly relevant in island systems, where arthropods often perform key ecosystem functions and where biodiversity trends remain less studied than in continental systems (Russell and Kueffer 2019).

Artificial light at night (ALAN) has been increasingly recognised as a driver of insect decline (Grubisic et al. 2018, Boyes et al. 2021, Koppenhagen et al. 2026, Schittko et al. 2026), with studies showing that it can affect insect abundance, behaviour, foraging, reproduction, as well as intra- and interspecific interactions and broader community structure (Owens and Lewis 2018, Grubisic and Grunsven 2021, Kehoe et al. 2022). Disruption of orientation mechanisms makes insects more susceptible to predation, often leading to prolonged flight around light sources and increased energetic costs (Langevelde et al. 2011, Owens and Lewis 2018). In addition, light pollution contributes to habitat fragmentation by creating barriers to movement and reducing landscape connectivity for nocturnal insects (Langevelde et al. 2011, Owens and Lewis 2018, Desouhant et al. 2019). Artificial lighting can alter dispersal, foraging and navigation behaviour, concentrating individuals around illuminated areas and disrupting movements between suitable habitats (Langevelde et al. 2011, Owens and Lewis 2018). These effects are particularly evident in species with limited dispersal ability, which may be less able to avoid illuminated areas or maintain connectivity between suitable habitats. In contrast, assessing the impacts of ALAN on highly mobile flying insects is often more challenging because light can influence movement behaviour over larger spatial scales, altering attraction, dispersal and activity patterns beyond the immediate vicinity of the light source (Owens and Lewis 2018, Grubisic and Grunsven 2021). Excessive public and road lighting at night can act as a strong attractant, creating ecological traps that may lead to mass mortality events (Eisenbeis 2006, Hölker et al. 2010). Illuminated areas may also function as sheltered microclimates within otherwise exposed landscapes, concentrating insects in high-risk zones (Grubisic and Grunsven 2021). Several mitigation measures have been proposed and implemented, including reducing light intensity and duration, limiting unnecessary lighting and modifying spectral composition to reduce ecological impacts (Hölker et al. 2010, Owens et al. 2020). Emerging evidence suggests that amber-coloured LEDs may be less harmful than white LEDs, although empirical support remains limited and taxon-specific, particularly for arthropods (Chevalier et al. 2025). Despite growing evidence of these impacts, studies assessing the effects of artificial light on arthropod communities remain scarce in Macaronesia.

Oceanic islands, such as Madeira, exhibit high levels of endemism and are particularly vulnerable to ecological disturbances, making baseline biodiversity data especially valuable for conservation planning (Borges et al. 2008, Boieiro et al. 2013). The Madeira Archipelago hosts a highly diverse and unique arthropod fauna, with more than 3,400 insect taxa, including over 750 endemic species, representing approximately 87% of all terrestrial arthropods recorded in the Archipelago (Borges et al. 2008). Coleoptera, Hymenoptera, Diptera, Hemiptera and Lepidoptera are the most diverse insect orders and account for the largest proportion of endemic taxa (Borges et al. 2008).

Despite recent advances, substantial knowledge gaps remain regarding the distribution, abundance and ecology of many Madeiran insect species (Florencio et al. 2021). Furthermore, only a small proportion of species have been assessed for extinction risk and discrepancies remain between species diversity and available conservation assessments (Casimiro 2017, BEST LIFE Programme 2024). These limitations hinder the establishment of robust biodiversity baselines and constrain effective conservation planning and management. Consequently, further biodiversity inventories and the publication of standardised species occurrence datasets remain essential for improving biodiversity knowledge, establishing baseline conditions and supporting the conservation of Madeiran terrestrial arthropods.

## General description

### Purpose

This dataset aims to provide a comprehensive inventory of terrestrial arthropods in Madeira’s Natura 2000 areas, supply georeferenced occurrence and abundance data and establish a baseline for future monitoring of nocturnal communities under artificial light exposure. The present dataset represents the baseline biodiversity survey component of LIFE Natura@night and was designed to document species occurrence prior to future assessments of ALAN impacts. Although multiple arthropod groups were surveyed, nocturnal Lepidoptera were subject to greater sampling and taxonomic identification effort, resulting in higher taxonomic resolution and making them the focus of the subsequent analyses. The present work provides the first large-scale standardised inventory of nocturnal arthropods across protected areas of Madeira Island, contributing baseline data for long-term biodiversity monitoring.

## Project description

### Title

Nocturnal arthropod occurrences across Natura 2000 protected areas of Madeira Island (Portugal)

### Personnel

Elisa Teixeira, Tânia Costa, Yasmin Redolosis, Azucena Martin, Cátia Gouveia, Virgílio Vieira and Paulo A.V. Borges planned the project and designed the sampling strategy; Elisa Teixeira, Tânia Costa, Adrià Bellmunt, Ana Amaral, Anna Cortés, Catarina Gomes, Elodie Crepin, Hornam Azanda, Lara Soares, Laura Sousa, Lúcio Cânovas, Matisse Vinotti, Manuel Contreras, Miguel Andrade, Miguel Pacheco, Renato Ornelas, Ricardo Reinolds, Simão Vieira, Thomas Quartier and Vítor Camacho participated in fieldwork. Elisa Teixeira, Tânia Costa and Virgílio Vieira identified the specimens. Elisa Teixeira, Mário Boieiro and Paulo A.V. Borges performed the biodiversity data curation in Darwin Core format.

### Study area description

To safeguard its unique biodiversity, Madeira Island hosts twelve terrestrial Natura 2000 sites covering ca. 35% of the island’s land area. Nine were selected for this study: SCI Cabo Girão (PTMAD0011), SCI Caniço de Baixo (PTMAD0012), SCI Machico (PTMAD0014), SCI Pico do Facho (PTMAD0015), SCI Porto Novo (PTMAD0013), SAC Pináculo (PTMAD0007), SAC Maciço Montanhoso Central da Ilha da Madeira (PTMAD0002), SPA/SAC Ponta de S. Lourenço (PTZPE0064/PTMAD0003) and SPA/SAC Laurissilva da Madeira (PTMAD0001). These sites encompass a broad range of habitat typologies representative of Madeira Island, including coastal habitats, dry shrublands, agricultural mosaics, exotic forests, native laurissilva forest and high-altitude mountain ecosystems. Other Natura 2000 terrestrial sites (Achadas da Cruz, Ilhéu da Viúva, Moledos–Madalena do Mar) were not sampled due to logistical constraints.

### Design description

A total of 48 sampling points were established across nine Natura 2000 sites on Madeira Island, separated by at least 250 m to maximise spatial independence between sampling locations. Sampling was conducted during two field campaigns, in late summer 2022 and spring 2023 (Table 1), resulting in a total of 68 sampling events. The second sampling campaign revisited selected locations that had yielded low numbers of specimens during the initial survey due to unfavourable weather conditions and/or reduced vegetation cover. Two additional sampling sites were also established in 2023 to improve coverage of the surveyed protected areas.

During each sampling event, arthropods were surveyed using two complementary methods: UV Heath light trapping and wine-rope bait trapping. Light trapping is a widely used and effective technique for sampling nocturnal Lepidoptera, the primary target group of the LIFE Natura@night project and enables standardised comparisons amongst sites. Furthermore, light traps have relatively local attraction ranges and, therefore, provide representative samples of local moth assemblages (Truxa and Fiedler 2012, Merckx and Slade 2014). Wine-rope bait traps were used as a complementary method to increase the detection of species that are weakly attracted or not attracted to artificial light (Fry and Waring 1996), thereby reducing potential sampling bias and improving sampling completeness.

Sampling was not conducted during periods of strong winds, rainfall or high humidity, as these conditions substantially reduce insect activity and trap efficiency. Environmental conditions, including temperature, relative humidity, cloud cover, wind intensity and sky darkness (using the Bortle scale), were recorded during each sampling event.

### Funding

The study was funded by LIFE Natura@night – Mitigating light pollution in Macaronesia (LIFE20 NAT/PT/001098).

## Sampling methods

### Sampling description

Terrestrial arthropods were sampled using Heath-type portable light traps complemented with wine-rope bait traps to maximise the detection of taxa with different attraction responses (Rossini et al. 2025). Heath-type light traps are widely used in standardised moth monitoring and biodiversity surveys due to their portability and effectiveness in attracting nocturnal Lepidoptera (Deijk et al. 2024). The five light traps consisted of a lidded bucket, equipped with a central funnel, UV-resistant methacrylate fins, a fluorescent lamp holder and a ballast connected to a 12 V battery. Illumination was provided by an Electrosect 18" 15 W UV-A BL fluorescent tube powered by a portable battery. All components were supplied by Entomopraxis. The complementary wine-rope bait method was deployed simultaneously using a thick rope soaked in a fermented sugar and wine solution and suspended from surrounding vegetation within the sampling area (Fry and Waring 1996). The bait rope remained exposed throughout the sampling period and arthropods attracted to the bait were collected directly from the rope using small plastic jars with lids (Fig. 1).

Sampling was conducted between approximately 21:00 h and 01:00 h under favourable weather conditions, characterised by the absence of precipitation, low wind speeds (0-10.7 m s⁻¹), an average air temperature of approximately 21°C and low moonlight levels. Field effort included:

- 2022: 13 sampling nights;
- 2023: 8 sampling nights.

### Quality control

Specimens were sorted and identified to the lowest possible taxonomic level using specialist literature, iNaturalist (https://www.inaturalist.org), the Global Biodiversity Information Facility (GBIF; https://www.gbif.org) and Lepiforum (https://lepiforum.org). Taxonomic nomenclature and species validation were checked against GBIF and Lepiforum. Lepidopterans were identified (or their identification confirmed) by a trained taxonomist (Virgílio Vieira). Taxa considered potential new records for Madeira were confidently identified to species level by specialists; however, they are referred to as potential new records because no prior published records were located during the literature review, although undocumented historical records cannot be entirely excluded.

### Step description

The taxonomy and nomenclature followed Borges et al. (2008) and the GBIF taxonomic backbone.

## Geographic coverage

### Description

Location: Madeira Island, Portugal.

Protected areas: nine Natura 2000 sites.

Sampling sites (Fig. 2) were distributed across habitat types including native vegetation, mixed vegetation, exotic vegetation, agricultural habitat and coastal vegetation (Fig. 3).

### Coordinates

32.638 and 32.83 Latitude; -16.698 and -17.032 Longitude.

## Taxonomic coverage

### Taxa included

**Table:** 

Rank	Scientific Name	Common Name
order	Araneae	Spiders
order	Blattodea	Cockroaches and termites
order	Coleoptera	Beetles
order	Dermaptera	Earwigs
order	Diptera	Hoverflies, blowflies and other flies
order	Ephemeroptera	Mayflies
order	Hemiptera	True bugs
order	Hymenoptera	Bees, bumblebees, wasps and ants
order	Isopoda	Woodlice
order	Julida	Millipedes
order	Lepidoptera	Butterflies and moths
order	Neuroptera	Lacewings and allies
order	Orthoptera	Grasshoppers and crickets
order	Psocodea	Booklice and barklice
order	Scutigeromorpha	House centipedes
order	Trichoptera	Caddisflies

## Temporal coverage

**Data range:** 2022-7-21 – 2023-6-19.

### Notes

The sampling periods were selected to coincide with periods of higher adult activity of nocturnal Lepidoptera in Madeira, based on previous field experience and the expert knowledge of the project taxonomist (Virgílio Vieira). Data were collected during two consecutive years: from 21 July 2022 to 30 August 2022 and from 19 April 2023 to 19 June 2023.

## Usage licence

### Usage licence

Creative Commons Public Domain Waiver (CC-Zero)

## Data resources

### Data package title

LIFE Natura@night baseline dataset of nocturnal terrestrial arthropods from Madeira Island, Portugal

### Resource link

https://doi.org/10.15468/kdfdga

### Alternative identifiers

https://www.gbif.org/dataset/115de336-e519-4fc8-9a60-28e601ce2876

### Number of data sets

2

### Data set 1.

#### Data set name

Event Table

#### Data format

Darwin Core

#### Character set

UTF-8

#### Download URL

https://ipt.gbif.pt/ipt/resource?r=arthropod_madeira_life_naturaatnight

#### Data format version

1.6

#### Description

The dataset was published in the Global Biodiversity Information Facility platform (GBIF) (Teixeira et al. 2026). The following data table includes all the records for which a taxonomic identification of the species was possible. The dataset submitted to GBIF is structured as a sample event dataset that has been published as a Darwin Core Archive (DwCA), which is a standardised format for sharing biodiversity data as a set of one or more data tables. The core data file contains 134 records (eventID). This IPT (Integrated Publishing Toolkit) archives the data and, thus, serves as the data repository. The data and resource metadata are available for download in the Portuguese GBIF Portal IPT (Teixeira et al. 2026).

**Data set 1.** 

Column label	Column description
eventID	Unique identifier of the sampling event within the dataset.
locationID	Identifier of the geographic location where the sampling event occurred.
stateProvince	Name of the administrative region.
islandGroup	Name of the island group or archipelago where the event took place.
country	Name of the country where the event occurred.
countryCode	Two-letter ISO code of the country (PT).
municipality	Municipality where the sampling event was conducted.
minimumElevationInMetres	Exact elevation of the sampling site above sea level, in metres.
decimalLongitude	Geographic longitude of the sampling location in decimal degrees (WGS84).
decimalLatitude	Geographic latitude of the sampling location in decimal degrees (WGS84).
geodeticDatum	Spatial reference system used for the geographic coordinates (WGS84).
coordinatePrecision	Precision of the geographic coordinates, in decimal degrees.
georeferenceSources	Source or method used to obtain the geographic coordinates.
coordinateUncertaintyInMetres	A horizontal distance (in metres) from a given dwc:decimalLatitude and dwc:decimalLongitude describing the smallest circle containing the whole of the dcterms:Location.
habitat	Habitat type where the sampling event was conducted.
Year	Four-digit year in which the sampling event occurred.
eventDate	Date or date range when the sampling event took place (ISO 8601 format).
samplingEffort	Description of the sampling effort applied during the event.
eventTime	The time or interval during which a dwc:Event occurred.
island	The name of the island on or near which the dcterms:Location occurs.
locality	Name of the specific location.
locationRemarks	Comments or notes about the dcterms:Location. Added specific locality description of the sampling site.
samplingProtocol	Method used to sample arthropods (e.g. Heath UV light trap, wine-rope bait trap).

### Data set 2.

#### Data set name

Occurrence Table

#### Data format

Darwin Core

#### Character set

UTF-8

#### Download URL

https://ipt.gbif.pt/ipt/resource?r=arthropod_madeira_life_naturaatnight

#### Data format version

1.6

#### Description

The dataset was published in the Global Biodiversity Information Facility platform, GBIF (Teixeira et al. 2026). The following data table includes all the records for which a taxonomic identification of the species was possible. The dataset submitted to GBIF is structured as a sample event dataset that has been published as a Darwin Core Archive (DwCA), which is a standardised format for sharing biodiversity data as a set of one or more data tables. The core data file contains 1939 records (occurrenceID). This IPT (Integrated Publishing Toolkit) archives the data and, thus, serves as the data repository. The data and resource metadata are available for download in the Portuguese GBIF Portal IPT (Teixeira et al. 2026).

**Data set 2.** 

Column label	Column description
eventID	Identifier of the sampling event to which the occurrence record is linked.
type	Nature of the record according to Darwin Core terminology.
licence	Usage licence assigned to the record (e.g. CC-BY 4.0).
institutionID	Globally unique identifier of the institution holding the record.
institutionCode	Code of the institution having custody of the data or specimens.
basisOfRecord	Specific nature of the data record (e.g. HumanObservation, PreservedSpecimen).
occurrenceStatus	Presence or absence status of the taxon at the sampling event.
occurrenceID	Unique identifier for the occurrence record.
organismQuantity	Number of individuals recorded for the taxon in the event.
organismQuantityType	Type of quantification used for organismQuantity (e.g. individuals).
establishmentMeans	Statement about whether the organism is native, introduced or of uncertain origin (biogeographic status).
degreeOfEstablishment	The degree to which a dwc:Organism survives, reproduces and expands its range at the given place and time.
recordedBy	Name(s) of the person(s) who recorded the occurrence.
identifiedBy	Name(s) of the person(s) who identified the taxon.
dateIdentified	Date when the taxon identification was performed.
kingdom	Highest taxonomic rank of the recorded organism.
phylum	Phylum to which the organism belongs.
class	Class to which the organism belongs.
order	Order to which the organism belongs.
family	Family to which the organism belongs.
scientificName	Full scientific name of the organism, including authorship when available.
genus	Genus name of the organism.
specificEpithet	Species epithet of the organism.
infraspecificEpithet	Infraspecific epithet, when applicable.
scientificNameAuthorship	Authorship information for the scientific name.
taxonRank	Lowest taxonomic rank of the recorded organism.

## Additional information

The dataset comprises 1,904 presence records and 35 absence records (organismQuantity = 0), corresponding to sampling events conducted using the wine-rope trap method. A total of 11,885 arthropod individuals were recorded. Of these, 200 taxa were identified to species or subspecies level (195 species and five subspecies), representing 6,028 individuals, whilst the remaining 5,857 individuals were assigned to higher taxonomic levels.

Taxon: Arthropoda. The dataset includes 200 taxa belonging to 16 terrestrial arthropod groups, 63 families and 160 genera. Lepidoptera was the dominant group, comprising 179 taxa (Fig. 4) and accounting for 59.7% of all recorded individuals (7,081 of 11,885 individuals; Fig. 5). Most Lepidoptera taxa were classified as native (38%) or endemic (35.2%), with introduced and migratory taxa representing 21.2% and 5.6% of the total, respectively (Fig. 6).

This study provides the first standardised baseline of the distribution and abundance of nocturnal Lepidoptera in Natura 2000 sites across central and eastern Madeira, establishing an important reference for future monitoring programmes and for identifying knowledge gaps, particularly concerning endemic species.

Although UV light trapping proved to be substantially more effective, recording 170 exclusive taxa, the complementary use of wine-rope bait trapping increased inventory completeness by detecting two species not recorded by light traps (*Periplaneta americana* (Linnaeus, 1758) *and Scoliopteryx libatrix* Linnaeus, 1758), in addition to the 28 taxa shared between methods. These findings demonstrate that different sampling techniques target distinct components of arthropod communities, likely reflecting behavioural differences in attraction to artificial light and fermented baits (Merckx and Slade 2014, Rossini et al. 2025). Consequently, despite contributing relatively few exclusive taxa, wine-rope bait trapping represents a valuable complementary tool for nocturnal biodiversity surveys. For future monitoring programmes, particularly in sites that have already been surveyed, standardised UV light trapping may provide the most efficient use of sampling effort and resources, whereas the combination of both methods may be preferable when the objective is to maximise inventory completeness and detect a broader range of taxa.

Several endemic moth species were recorded only rarely and/or at a limited number of sampling sites. Particularly noteworthy were *Blastobasis bassii*, *Blastobasis laurisilvae*, *Blastobasis wollastoni*, *Blepharita inexspectata*, *Herbulotina maderae*, *Mythimna serradaguae*, *Noctua teixeirai*, *Xenochlorodes magna* and *Zelleria wolffi*, all of which were represented by very few individuals. In addition, *Agrotis atrux*, *Carposina anopta*, *Eupithecia latipennata* and *Eupithecia rosai* showed restricted occurrence patterns within the study area.

These low detection rates may reflect naturally small populations, restricted distributions, specialised habitat requirements, phenological constraints or limitations associated with the sampling methods employed, which may provide only a partial representation of local arthropod populations (Rossini et al. 2025). Nevertheless, their apparent rarity highlights the need for continued monitoring and targeted ecological studies to better understand their distribution, population status and conservation requirements. Particular attention should be given to *Noctua teixeirai*, one of the least frequently recorded Madeiran endemics in the present survey and potentially amongst the most vulnerable.

The survey provided new insights into the arthropod fauna of Madeira, including two potential new records for the Archipelago (*Blastesthia tessulatana* and *Heterotoma planicornis*), a possible first confirmed record (*Eudonia mercurella*) and the re-confirmation of a previously doubtful taxon (*Blastobasis phycidella*). However, some of these records require further taxonomic validation. In particular, the identification of *E. mercurella* must be confirmed through the presence of its larval stages or a DNA analysis complementing the comparative examination of the genitalia, due to its similarity to endemic *E. scoriella*, while *B. phycidella* must be compared with morphologically similar Madeiran congeners. Likewise, the occurrence of *Idaea carvalhoi* remains provisional, pending DNA analysis and/or genitalic examination to rule out confusion with the endemic *Idaea atlantica*. These findings highlight the importance of combining long-term biodiversity monitoring with detailed taxonomic studies to improve our understanding of the diversity of terrestrial arthropods in Madeira. The dataset also documents the occurrence of *Hipparchia maderensis*, the only butterfly recorded during the study and one of the Archipelago’s most characteristic butterfly taxa.

The data generated here provide a valuable foundation for future conservation assessments, long-term monitoring of community change and the identification of priority taxa and areas for conservation action. Furthermore, artificial light at night should be considered in future conservation and management strategies, as light pollution can disrupt ecological interactions, including pollination processes and predator–prey dynamics, potentially affecting ecosystem functioning and the success of ecological restoration initiatives.

## Figures and Tables

**Figure 1.**
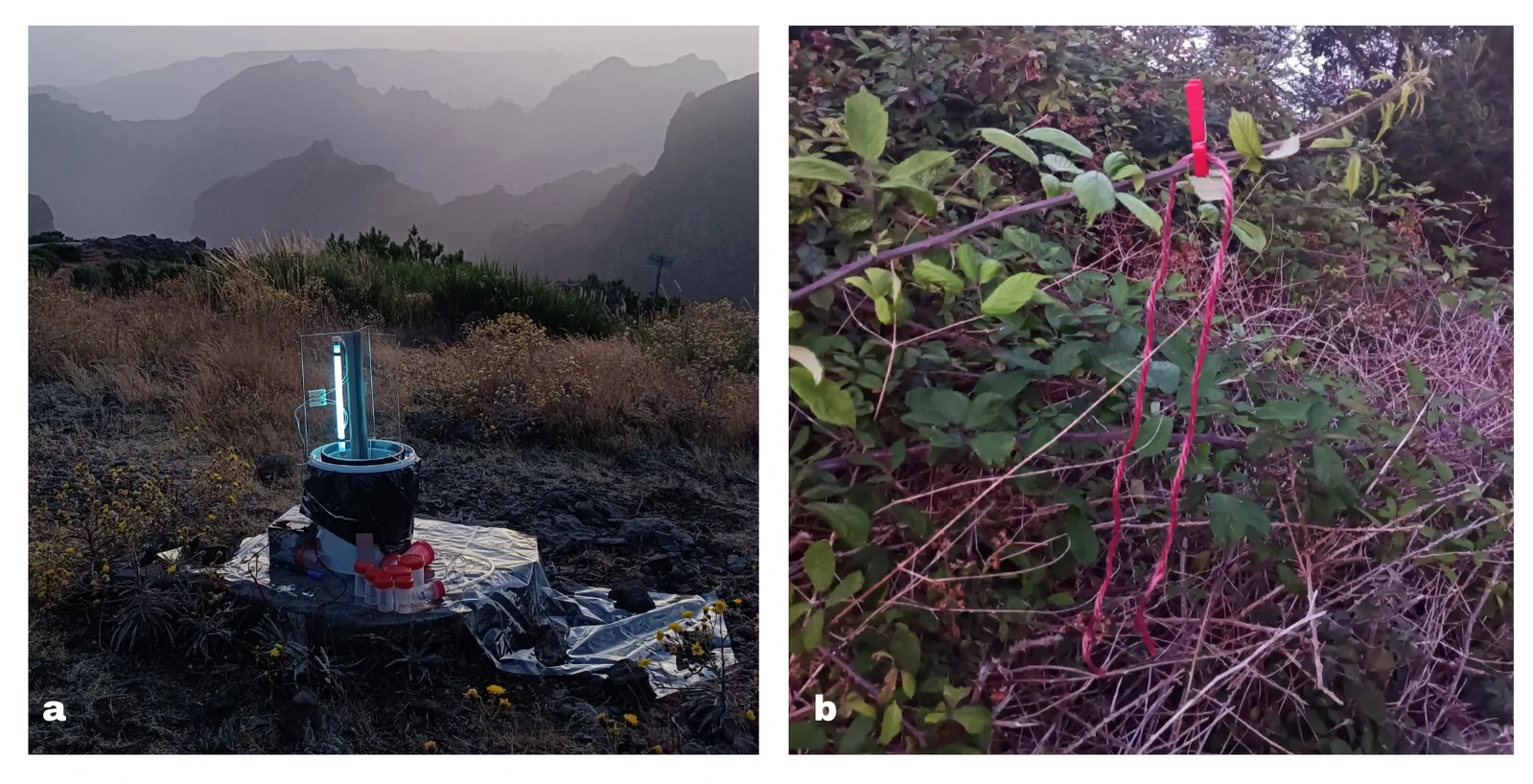
Methodologies employed simultaneously in this study. **a** UV Heath light trap; **b** Wine-rope bait trap (photos by Elisa Teixeira).

**Figure 2.**
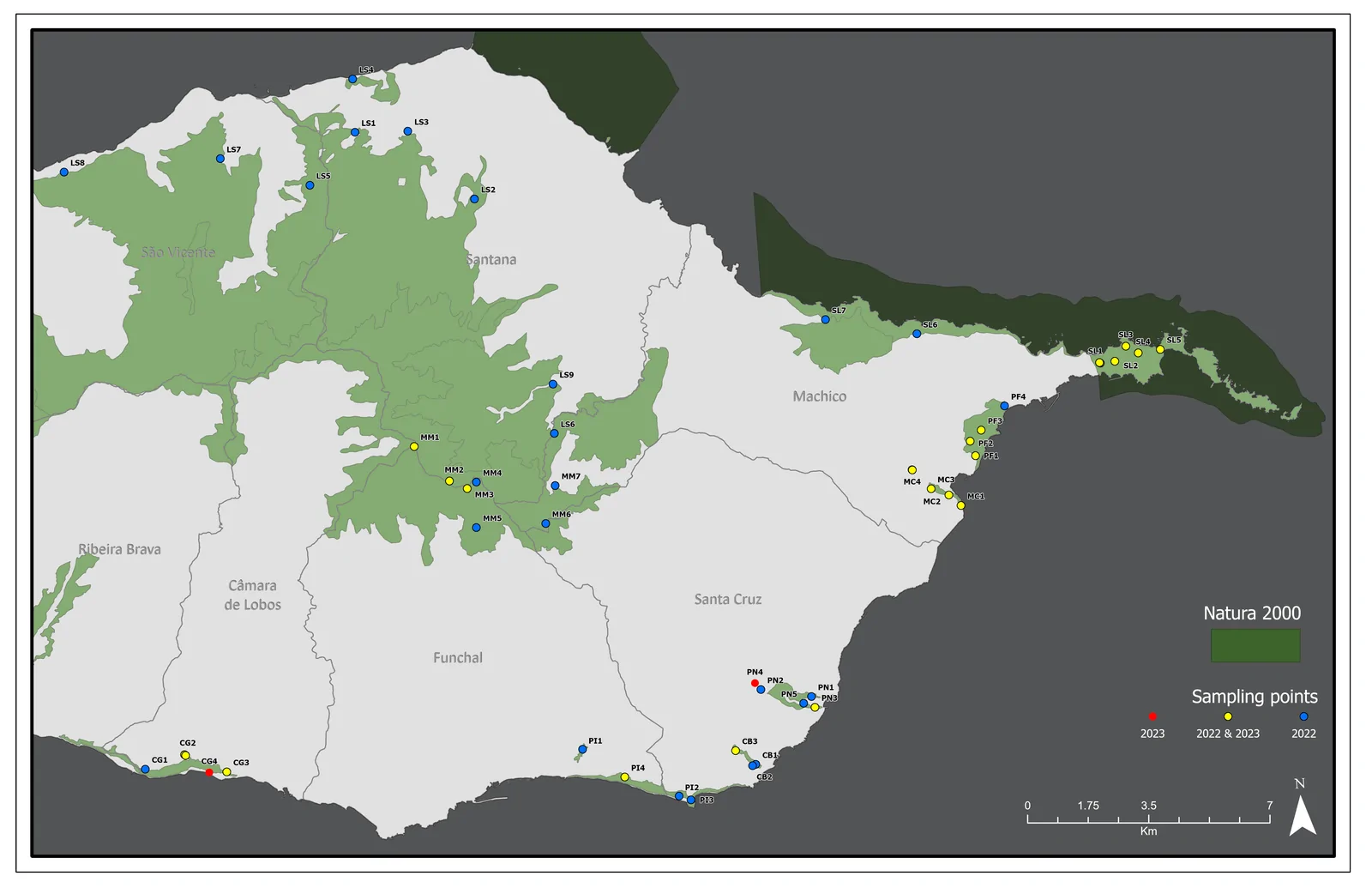
Sampling sites across Natura 2000 sites of Madeira Island. Authorship: Fernando Chacon (SPEA BirdLife).

**Figure 3.**
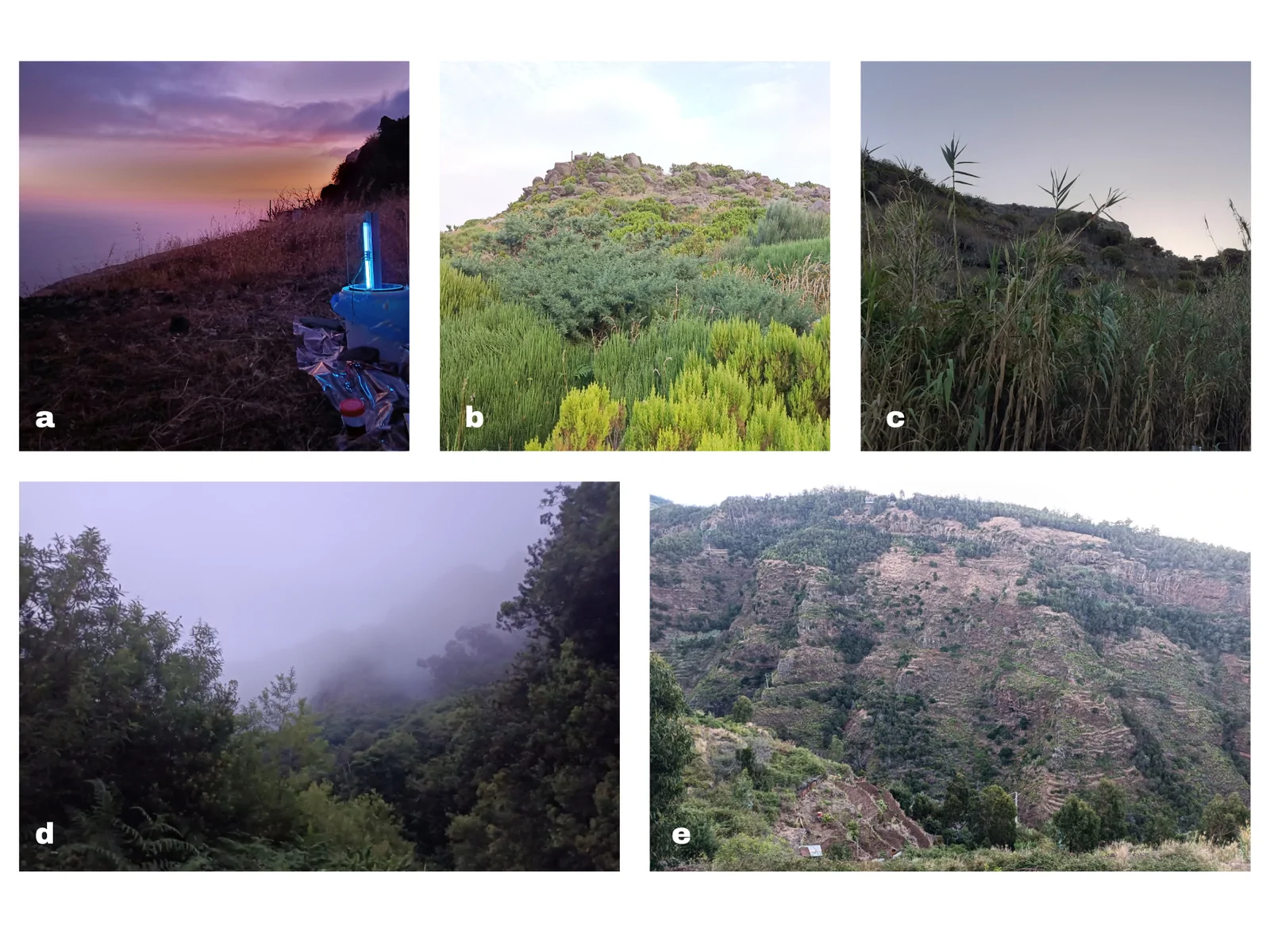
The five main habitat types sampled in this study. **a** Coastal vegetation in Cabo Girão (photo by Tânia Costa); **b** Mixed vegetation in Maciço Montanhoso Central da Ilha da Madeira (photo by Tânia Costa); **c** Exotic vegetation in Caniço de Baixo (photo by Laura Sousa); **d** Native vegetation in Laurissilva da Madeira (photo by Elisa Teixeira); **e** Agricultural habitat in Porto Novo (photo by Elisa Teixeira).

**Figure 4.**
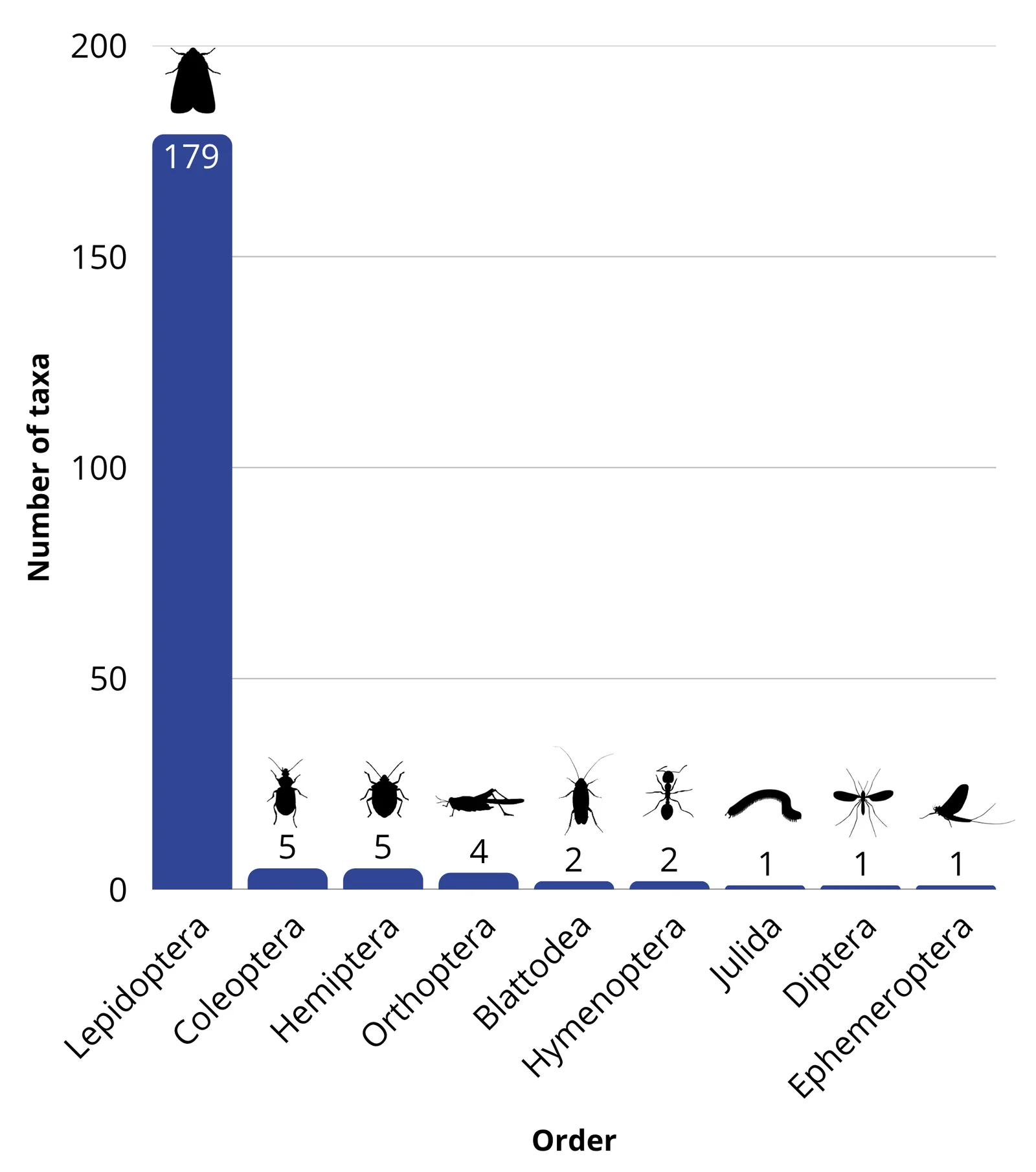
Number of taxa (species and subspecies) of arthropods identified in the dataset, by order (n = 200 taxa). All arthropod silhouettes were obtained from Phylopic (www.phylopic.org).

**Figure 5.**
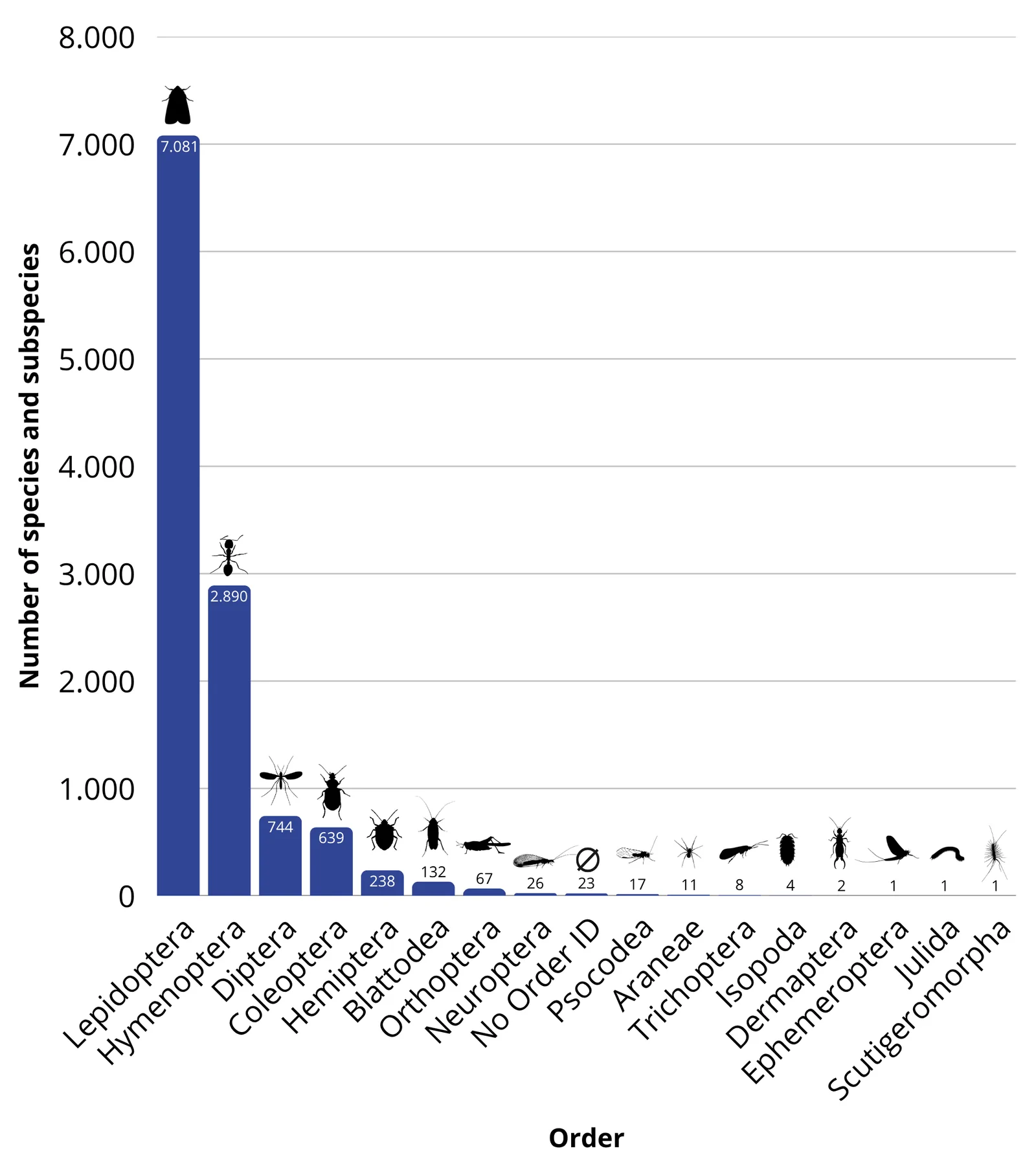
Arthropod orders represented in the dataset and their respective percentages of individuals (n = 11,885 individuals). All arthropod silhouettes were obtained from Phylopic (www.phylopic.org).

**Figure 6.**
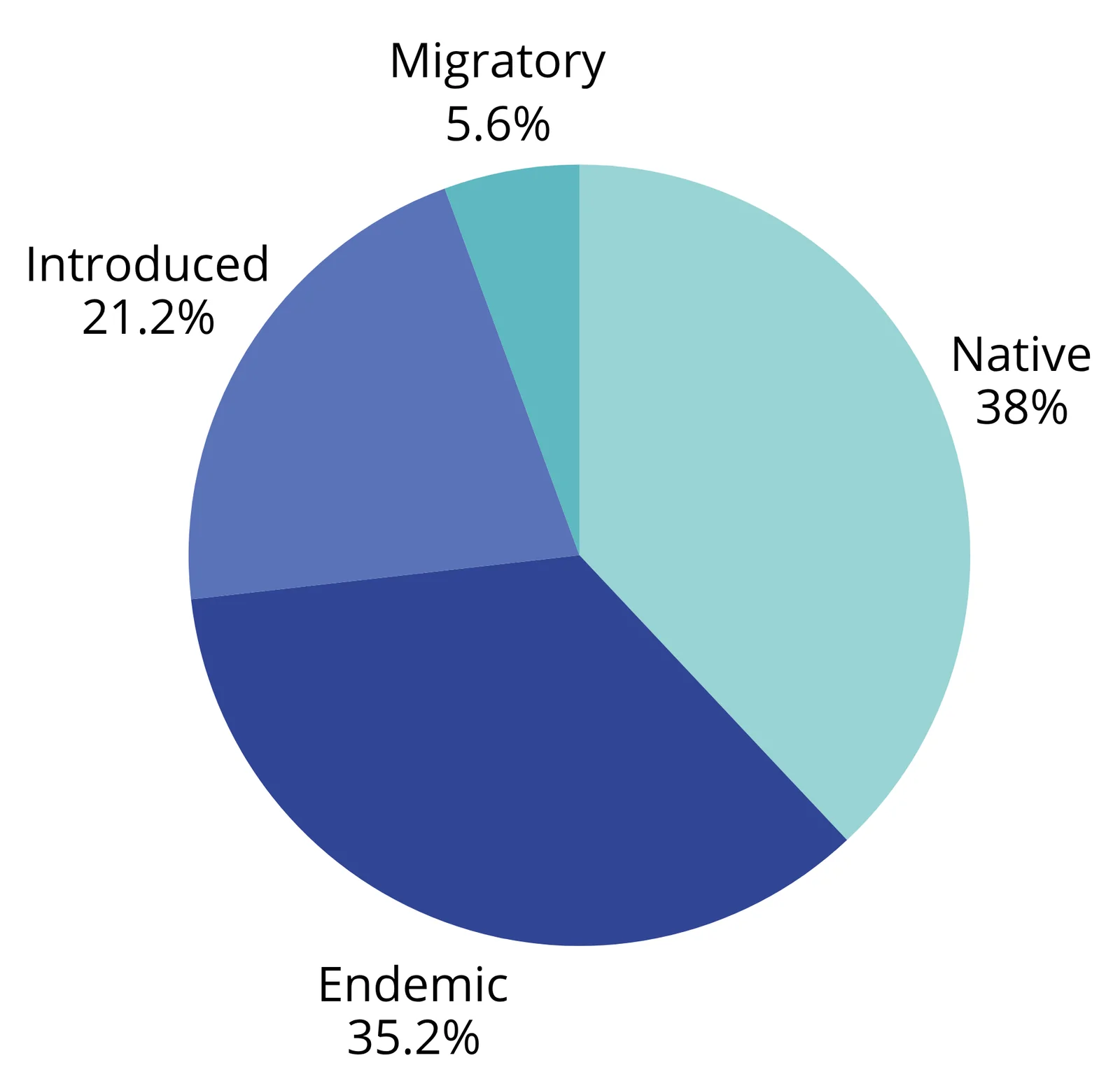
Relative proportion (%) of Lepidoptera taxa by biogeographic status (Native, Endemic, Introduced and Migratory) (n = 179 taxa [species and subspecies]).

**Table 1.** List of the study sites, including their geographic coordinates (decimal degrees, WGS84) and habitat type. The sampling years in each location are also indicated.

**Protected area**	**Site name**	**Habitat type**	**Latitude**	**Longitude**	**Years sampled**
SAC Maciço Montanhoso Central da Ilha da Madeira (PTMAD0002)	Pico do Areeiro	Mixed vegetation	32.734030	-16.929285	2022, 2023
SAC Maciço Montanhoso Central da Ilha da Madeira (PTMAD0002)	Pico do Areeiro Sul	Native vegetation	32.724525	-16.919077	2022, 2023
SAC Maciço Montanhoso Central da Ilha da Madeira (PTMAD0002)	Observatório de Astronomia da Madeira	Mixed vegetation	32.722329	-16.913771	2022, 2023
SAC Maciço Montanhoso Central da Ilha da Madeira (PTMAD0002)	Casa de Abrigo das Sorveiras	Mixed vegetation	32.723849	-16.910768	2022
SAC Maciço Montanhoso Central da Ilha da Madeira (PTMAD0002)	Chão da Lagoa	Mixed vegetation	32.712116	-16.911606	2022
SAC Maciço Montanhoso Central da Ilha da Madeira (PTMAD0002)	Poiso	Exotic vegetation	32.712072	-16.890250	2022
SAC Maciço Montanhoso Central da Ilha da Madeira (PTMAD0002)	Chão das Feiteiras	Native vegetation	32.721756	-16.886615	2022
SAC Pináculo (PTMAD0007)	Rua Conde Carvalhal, São Gonçalo	Exotic vegetation	32.653039	-16.883012	2022
SAC Pináculo (PTMAD0007)	Praia do Garajau	Coastal vegetation	32.639470	-16.854200	2022
SAC Pináculo (PTMAD0007)	Cristo Rei do Garajau	Coastal vegetation	32.638320	-16.850630	2022
SAC Pináculo (PTMAD0007)	Miradouro do Pináculo	Coastal vegetation	32.645216	-16.870606	2022, 2023
SAC/SPA Ponta de S. Lourenço (PTZPE0064/PTMAD0003)	Miradouro do Cabo de Larano	Coastal vegetation	32.760442	-16.800441	2022
SAC/SPA Ponta de S. Lourenço (PTZPE0064/PTMAD0003)	Boca do Risco	Native vegetation	32.755353	-16.772588	2022
SAC/SPA Ponta de S. Lourenço (PTZPE0064/PTMAD0003)	Prainha do Caniçal	Coastal vegetation	32.745081	-16.716942	2022, 2023
SAC/SPA Ponta de S. Lourenço (PTZPE0064/PTMAD0003)	Quinta do Lorde	Exotic vegetation	32.745232	-16.712192	2022, 2023
SAC/SPA Ponta de S. Lourenço (PTZPE0064/PTMAD0003)	Ponta do Rosto	Coastal vegetation	32.748920	-16.708503	2022, 2023
SAC/SPA Ponta de S. Lourenço (PTZPE0064/PTMAD0003)	Miradouro do Caniçal	Coastal vegetation	32.746975	-16.704783	2022, 2023
SAC/SPA Ponta de S. Lourenço (PTZPE0064/PTMAD0003)	Ponta de São Lourenço	Coastal vegetation	32.747586	-16.697930	2022, 2023
SCI Cabo Girão (PTMAD0011)	Miradouro da Quinta Grande	Coastal vegetation	32.654730	-17.017920	2022
SCI Cabo Girão (PTMAD0011)	Norte do Miradouro do Cabo Girão	Exotic vegetation	32.657830	-17.005600	2022, 2023
SCI Cabo Girão (PTMAD0011)	Teleférico do Rancho	Coastal vegetation	32.652760	-16.992910	2022, 2023
SCI Cabo Girão (PTMAD0011)	Cabo Girão	Coastal vegetation	32.652897	-16.998274	2023
SCI Caniço de Baixo (PTMAD0012)	Sudeste Ribeira do Caniço de Baixo	Exotic vegetation	32.646481	-16.830016	2022
SCI Caniço de Baixo (PTMAD0012)	Sudoeste Ribeira do Caniço de Baixo	Agricultural habitats	32.646165	-16.831049	2022
SCI Caniço de Baixo (PTMAD0012)	Noroeste Ribeira do Caniço de Baixo	Mixed vegetation	32.650358	-16.835974	2022, 2023
SCI Machico (PTMAD0014)	Miradouro e Baloiço do Coração	Coastal vegetation	32.710282	-16.762143	2022, 2023
SCI Machico (PTMAD0014)	Caminho das Voltinhas	Coastal vegetation	32.713188	-16.765672	2022, 2023
SCI Machico (PTMAD0014)	Caminho da Portelinha	Coastal vegetation	32.715063	-16.771013	2022, 2023
SCI Machico (PTMAD0014)	Boa Vista	Agricultural habitats	32.720227	-16.776491	2022, 2023
SCI Pico do Facho (PTMAD0015)	Sul do Miradouro do Pico do Facho	Coastal vegetation	32.722931	-16.756797	2022, 2023
SCI Pico do Facho (PTMAD0015)	Pico do Facho Este	Coastal vegetation	32.726795	-16.758175	2022, 2023
SCI Pico do Facho (PTMAD0015)	Pico do Facho Oeste	Coastal vegetation	32.729450	-16.754515	2022, 2023
SCI Pico do Facho (PTMAD0015)	Ribeira do Natal	Coastal vegetation	32.735350	-16.746880	2022
SCI Porto Novo (PTMAD0013)	Vale Este do Porto Novo	Agricultural habitats	32.663163	-16.811641	2022
SCI Porto Novo (PTMAD0013)	Forte do Porto Novo	Coastal vegetation	32.660353	-16.810775	2022, 2023
SCI Porto Novo (PTMAD0013)	Vale Oeste do Porto Novo	Coastal vegetation	32.661545	-16.814197	2022
SCI Porto Novo (PTMAD0013)	Levada do Caniço	Mixed vegetation	32.665770	-16.827098	2022
SCI Porto Novo (PTMAD0013)	Levada do Caniço Norte	Mixed vegetation	32.667513	-16.828819	2023
SPA/SAC Laurissilva da Madeira (PTMAD0001)	Rua do Cabeço, São Jorge	Mixed vegetation	32.816147	-16.941673	2022
SPA/SAC Laurissilva da Madeira (PTMAD0001)	Sítio do Lombo Antão Alves, Ilha	Mixed vegetation	32.797105	-16.906123	2022
SPA/SAC Laurissilva da Madeira (PTMAD0001)	Levada do Rei, São Jorge	Mixed vegetation	32.815641	-16.925386	2022
SPA/SAC Laurissilva da Madeira (PTMAD0001)	Terras de Fora, São Jorge	Coastal vegetation	32.829968	-16.941387	2022
SPA/SAC Laurissilva da Madeira (PTMAD0001)	Lamaceiros, São Vicente	Native vegetation	32.803205	-16.956560	2022
SPA/SAC Laurissilva da Madeira (PTMAD0001)	Cabo da Ribeira, Boaventura	Native vegetation	32.811440	-16.983625	2022
SPA/SAC Laurissilva da Madeira (PTMAD0001)	Fajã da Areia, São Vicente	Mixed vegetation	32.810417	-17.031940	2022
SPA/SAC Laurissilva da Madeira (PTMAD0001)	Ribeiro Frio	Mixed vegetation	32.735230	-16.885938	2022
SPA/SAC Laurissilva da Madeira (PTMAD0001)	Fajã da Nogueira	Mixed vegetation	32.748015	-16.885391	2022
